# Nanoflower-like CuCo_2_S_4_ with Bimetallic Synergy as High-Performance Bifunctional Electrocatalyst for Polysulfide/Iodide Redox Flow Batteries

**DOI:** 10.3390/ma19132839

**Published:** 2026-07-03

**Authors:** Shuo Liu, Renyi Wei, Jingwen Zhang, Xiaoxin Dan, Mingying Chen, Wenxian Liu, Jia He, Xijun Liu

**Affiliations:** 1 School of Energy and Chemical Engineering, Tianjin Renai College, Tianjin 301636, China; liushuo@nankai.edu.cn; 2 MOE Key Laboratory of New Processing Technology for Nonferrous Metals and Materials, Guangxi Key Laboratory of Processing for Non-Ferrous Metals and Featured Materials, School of Resources, Environment and Materials, Guangxi University, Nanning 530004, China; 3 College of Materials Science and Engineering, Zhejiang University of Technology, Hangzhou 310014, China; 4 Institute for School of Chemistry and Chemical Engineering, Tianjin University of Technology, Tianjin 300384, China

**Keywords:** polysulfide/iodide redox flow batteries, nanoflower-like structure, bimetallic synergy, bifunctional electrocatalyst

## Abstract

With the rapid development of grid-scale energy storage, aqueous polysulfide/iodide redox flow batteries (SIFBs) have attracted extensive attention owing to their low cost, high safety, and suitable output voltage. However, the sluggish redox kinetics of iodine and polysulfide couples and the severe shuttle effect seriously restrict their performance. Here, an ultrathin nanoflower-like CuCo_2_S_4_ electrocatalyst supported on graphite felt (GF) is rationally designed and synthesized via a hydrothermal method combined with high-temperature sulfurization. Benefiting from the unique open nanoflower structure, abundant multivalent metal sites, and strong Cu–Co bimetallic synergy, the as-prepared CuCo_2_S_4_ exhibits excellent adsorption capacity for polysulfide and polyiodide intermediates, small redox peak potential separation, and low charge transfer resistance. When applied in SIFBs, the CuCo_2_S_4_ electrode delivers a remarkably low voltage gap of 0.29 V at 20 mA cm^−2^, stable energy efficiency of 62–66% over 50 cycles, and superior long-term cycling stability with high energy efficiency above 70% after 400 cycles. This work provides an effective strategy for constructing high-efficiency bifunctional electrocatalysts toward high-performance and long-life SIFBs for large-scale energy storage applications.

## 1. Introduction

With the accelerating penetration of wind and solar power, grid-scale energy storage has become indispensable for stabilizing power fluctuation and improving energy utilization efficiency [1,2]. Among various electrochemical energy storage technologies, aqueous redox flow batteries (RFBs) are regarded as one of the most promising candidates owing to their high safety, decoupled power and capacity design, long cycle life, and low-cost potential [3,4,5]. In particular, polysulfide/iodide flow batteries (SIFBs) have attracted extensive attention due to the high solubility of active species, low material cost, suitable output voltage, and environmental friendliness. Therefore, SIFBs show great application prospects in medium- and long-term energy storage scenarios [6,7,8,9].

However, the practical application of SIFBs is severely restricted by two critical bottlenecks [10,11,12]. On the cathode side, the redox kinetics of the I^−^/I_3_^−^ couple is intrinsically sluggish, leading to large electrochemical polarization and low voltage efficiency [13,14]. On the anode side, the multi-step electron transfer of polysulfide species (S^2−^/S_x_^2−^) is kinetically slow, and the severe shuttle effect causes serious active substance loss, low Coulombic efficiency, and rapid capacity decay [15,16]. To overcome these problems, developing highly efficient bifunctional electrocatalysts that can simultaneously accelerate the redox kinetics of iodine and polysulfide couples and suppress the shuttle effect has become the core strategy to improve the overall performance of SIFBs [7,17,18].

In recent years, transition metal sulfides have been widely investigated as high-performance electrocatalysts for SIFBs. Among them, cobalt-based sulfides exhibit excellent catalytic activity for both iodine and polysulfide redox reactions due to their abundant active sites, suitable electronic structure, and strong chemical adsorption ability [19,20,21]. Nevertheless, monometallic cobalt sulfides still suffer from insufficient active sites, poor electrical conductivity, and structural instability under long-term cycling [22,23,24]. Constructing bimetallic spinel sulfides provides an effective approach to break through these limitations. The synergistic effect between different metal sites can optimize the electronic structure, enhance charge transfer, generate more exposed active centers, and improve structural stability. Especially, CuCo_2_S_4_ with a typical spinel structure possesses adjustable valence states (Cu^+^/Cu^2+^, Co^2+^/Co^3+^), rich defects, high conductivity, and strong adsorption affinity for polysulfide and iodide intermediates, making it an ideal bifunctional electrocatalyst for SIFBs [25,26,27,28].

In this work, ultrathin nanoflower-like CuCo_2_S_4_ grown on graphite felt (GF) was rationally designed and synthesized via a facile hydrothermal method combined with high-temperature sulfurization. The unique nanoflower morphology significantly increases the specific surface area, exposes abundant active sites, and facilitates electrolyte infiltration and ion diffusion. Meanwhile, the bimetallic synergistic effect between Cu and Co optimizes the adsorption behavior of key intermediates, accelerates interfacial charge transfer, and effectively suppresses the shuttle effect. When employed as a bifunctional electrocatalyst for SIFBs, CuCo_2_S_4_ remarkably reduces the reaction polarization, enhances the energy efficiency and rate capability, and prolongs the cycling stability. This work demonstrates that the spinel-structured bimetallic sulfide is a highly efficient electrocatalyst for SIFBs and provides a feasible strategy for developing high-performance electrode materials for advanced low-cost and long-life redox flow batteries.

## 2. Experimental Section

### 2.1. Synthesis of CuCo_2_S_4_ Nanoflowers on GF and CoS_1.097_

In a typical synthesis, 0.7600 g thiourea, 0.188 g Cu(NO_3_)_2_ (Macklin, from Shanghai, China), and 1.1640 g Co(NO_3_)_2_·6H_2_O were dissolved in 75 mL deionized water under continuous stirring to form a homogeneous solution. Thiourea and Co(NO_3_)_2_·6H_2_O are purchased from Sinopharm Chemical Reagent (from Shanghai, China). The solution together with a piece of pre-cleaned graphite (Beijing Jinglong Special Carbon Technology, from Beijing, China) felt was transferred into a 100 mL Teflon-lined stainless-steel autoclave and heated at 200 °C for 20 h (purchased from Suzhou Shenghua Instrument Technology, from Suzhou, China). After cooling to room temperature naturally, the product was washed with deionized water and ethanol several times and dried at 60 °C overnight. Subsequently, the as-obtained product and 0.64 g sublimed sulfur (Beijing Hanlongda Technology Development, from Beijing, China) were placed at the downstream and upstream of a porcelain boat in a tube furnace (Hefei Kejing Materials Technology, from Hefei, China), respectively. After annealing at 400 °C for 2 h under a nitrogen atmosphere, the final CuCo_2_S_4_ nanoflowers supported on graphite felt were obtained and denoted as CuCo_2_S_4_.

For comparison, CoS_1.097_ was synthesized via the same hydrothermal and sulfurization procedure except that Cu(NO_3_)_2_ was removed from the precursor solution. The final product was denoted as CoS_1.097_.

### 2.2. Electrochemical Measurements

Cyclic voltammetry (CV) measurements were performed on a CHI760D electrochemical workstation with a typical three-electrode system, where a platinum sheet, a saturated calomel electrode (Hg/Hg_2_Cl_2_), and the as-prepared catalyst or graphite felt (GF) were used as the counter, reference, and working electrodes, respectively. For the preparation of the working electrode, 10 mg of powder catalyst was dispersed in a mixture of 500 μL deionized water and 500 μL isopropyl alcohol, followed by the addition of 20 μL of 5 wt% Nafion solution, and the mixture was ultrasonicated for 30 min to form a homogeneous ink, which was then uniformly drop-cast onto a 3 mm-diameter glassy carbon electrode and dried at room temperature, while bare GF was directly tested as a control working electrode. CV tests were carried out in 0.06 M Na_2_S + 0.02 M S + 0.5 M NaCl and 0.1 M NaI_3_ + 0.5 M NaCl aqueous solutions at a fixed scan rate of 50 mV·s^−1^ or a series of gradient scan rates from 10 to 100 mV·s^−1^ (10, 20, 30, 40, 50, 60, 70, 80, 90, 100 mV·s^−1^).

Visual adsorption tests were conducted by immersing electrode materials with the same area into 20 mL aqueous solutions of NaI_3_ (prepared with 6 mM NaI and 1.5 mM I_2_) and Na_2_S_x_ (15 mM Na_2_S_2_) respectively, followed by continuous stirring at room temperature for 24 h to ensure sufficient adsorption and equilibrium of active species on the material surfaces [14,21]. UV–vis spectrophotometry was then used to record the absorption spectra of the solutions after adsorption, and the adsorption performance of different electrodes was quantitatively compared by analyzing the variation in characteristic peak intensity.

Detailed structural and component parameters of the flow cell are provided in the Supplementary Materials. The optical photograph of the assembled single flow cell with complete cell components is displayed in Figure S6. Flow battery performance tests were carried out on a NEWARE battery testing system (Shenzhen Neware Electronics Co., Ltd., Shenzhen, China). Galvanostatic charge–discharge tests were conducted at various current densities. The charging process was terminated by capacity cutoff corresponding to 100%, 50%, and 10% state of charge (SOC), with an upper voltage limit of 2.0 V for overcharge protection. The discharging process was controlled by both capacity cutoff (100%, 50%, and 10% SOC) and a low-voltage cutoff of 0.1 V. The catholyte was 2.0 M NaI + 0.5 M I_2_, and the anolyte was 2.0 M Na_2_S_2_. The electrolyte flow rate was fixed at 10 mL min^−1^ during all measurements. The theoretical capacity was calculated based on the catholyte (iodine component), using 5 mL of 2.0 M NaI + 0.5 M I_2_, yielding a nominal capacity of 134 mAh.

Electrochemical impedance spectroscopy (EIS) tests of the assembled full cell were performed using a CHI760D electrochemical workstation. Before the test, the assembled full cell was connected to the electrolyte circulation system, and the peristaltic pump was turned on to continuously circulate the cathodic and anodic electrolytes inside the cell for 5 min. This operation ensured that both the surface and internal pores of the graphite felt electrode were fully wetted by the electrolyte, eliminating poor interfacial contact caused by insufficient wetting. After complete electrode wetting, the electrolyte circulation was stopped, and the EIS measurement was immediately conducted. The test parameters were set as follows: the frequency scanning range was from 0.1 Hz to 100 kHz, the amplitude of the AC excitation signal was 10 mV, and the test was carried out at the open-circuit potential. By collecting the impedance response at different frequencies, kinetic information including ohmic resistance, charge transfer resistance and diffusion impedance of the cell can be further analyzed.

## 3. Results and Discussion

### 3.1. Structure and Morphology Characterization

**Figure 1:**
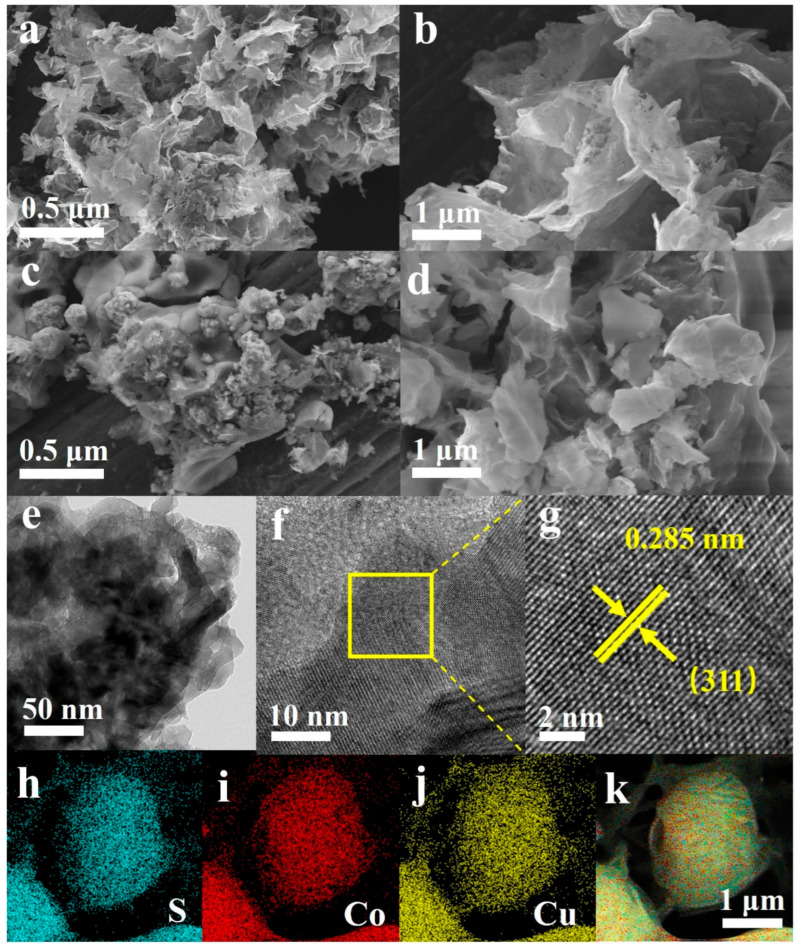
(**a**,**b**) and (**c**,**d**) are SEM images of CuCo_2_S_4_, CoS_1.097_ electrodes, respectively. (**e**–**g**) and (**h**–**k**) are TEM and EDX mapping images of the CuCo_2_S_4_ electrode, respectively.

The morphology and microstructure of CoS_1.097_ and CuCo_2_S_4_ were observed by scanning electron microscopy (SEM). As presented in Figure 1a,b, CuCo_2_S_4_ exhibits a unique ultrathin nanoflower-like hierarchical architecture assembled by interconnected 2D nanosheets. In sharp contrast, CoS_1.097_ shows severe agglomeration with irregular bulk structures, resulting in insufficient exposed active sites and blocked ion transport channels (Figure 1c,d). The 3D open porous network not only enlarges the specific surface area but also facilitates electrolyte penetration and rapid charge transfer. Such favorable morphology is highly beneficial for exposing abundant active sites and accelerating redox kinetics of polysulfide and iodide species in SIFBs.

Transmission electron microscopy (TEM) and high-resolution TEM (HRTEM) were further employed to reveal the detailed crystalline structure of CuCo_2_S_4_. As depicted in Figure 1e,f, the nanoflower morphology is clearly observed, which consists of ultrathin and curly nanosheets. The HRTEM image (Figure 1g) displays distinct lattice fringes with an interplanar spacing of 0.285 nm, corresponding to the (311) crystal plane of spinel CuCo_2_S_4_. Moreover, the energy-dispersive X-ray spectroscopy (EDX) elemental mappings (Figure 1h–k) demonstrate that Cu, Co, and S elements are homogeneously distributed throughout the entire nanoflower structure without obvious aggregation or segregation. The uniform elemental distribution confirms the successful formation of CuCo_2_S_4_ spinel structure and indicates strong electronic interaction between Cu and Co species, which is crucial for boosting the bifunctional electrocatalytic performance toward I^−^/I_3_^−^ and S^2−^/S_x_^2−^ redox reactions.

**Figure 2:**
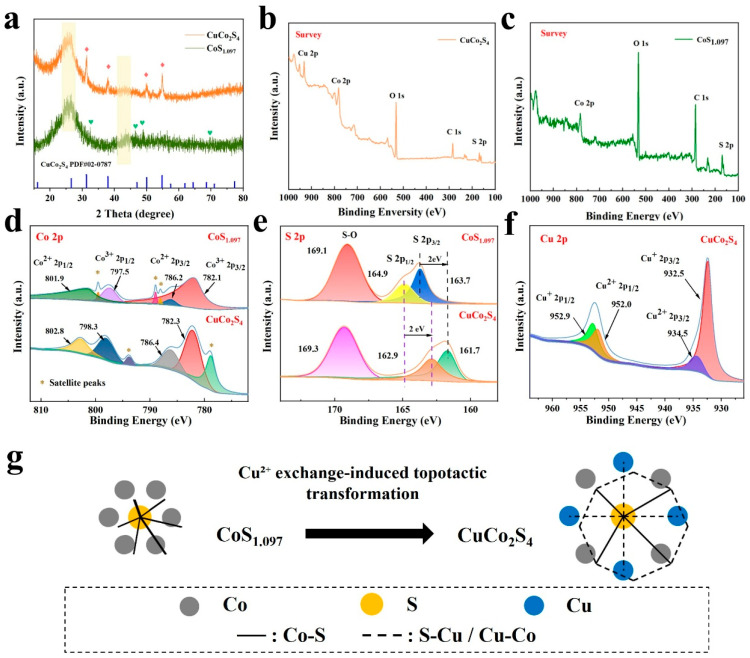
(**a**) XRD plots of different electrodes. XPS survey spectra of CuCo_2_S_4_ (**b**) and CoS_1.097_ (**c**). Co 2p (**d**) and S 2p (**e**) fine spectra of CuCo_2_S_4_ and CoS_1.097_. (**f**) Cu 2p fine spectrum of CuCo_2_S_4_. (**g**) Schematic diagram of the coordination structure showing the topological transformation of CoS_1.097_ to CuCo_2_S_4_ induced by Cu^2+^ exchange.

The crystal phase and purity of as-prepared samples were then characterized by X-ray diffraction (XRD), as displayed in Figure 2a. For the CoS_1.097_ sample, several weak diffraction peaks located at 32.07°, 47.02°, and 49.10° are well indexed to the hexagonal phase of CoS_1.097_ (PDF#19-0366). After Cu introduction and further sulfurization treatment, the diffraction peaks shift to 31.33°, 37.97°, 49.99°, and 54.75°; these peaks correspond to the (311), (400), (511), and (440) crystal planes of CuCo_2_S_4_, respectively, which match well with the cubic spinel phase of CuCo_2_S_4_ (PDF#02-0787) [25]. No peaks belonging to impurities such as CuS, CoS_2_, or other oxides are detected, confirming the successful preparation of pure-phase CuCo_2_S_4_. Moreover, these peak positions are shifted compared to those of metallic Co and Cu, indicating the successful formation of a pure sulfide spinel phase rather than the retention of metallic Co or Cu [26]. Meanwhile, the broad peak at around 26° corresponds to the characteristic peak of graphite felt (GF), which is consistent with previous reports [7].

X-ray photoelectron spectroscopy (XPS) was performed to analyze the surface chemical composition and valence states of CuCo_2_S_4_ and CoS_1.097_. The survey spectrum confirms the existence of Cu, Co, S, and C elements without other impurities (Figure 2b,c). For the high-resolution Co 2p spectra (Figure 2d), two pairs of fitting peaks corresponding to Co^2+^ and Co^3+^ are observed in both samples [29,30]. After Cu introduction, the binding energy of Co 2p positively shifts, indicating electron transfer from Co to Cu and strong electronic interaction in CuCo_2_S_4_ [31,32]. As for S 2p spectra (Figure 2e), the peaks corresponding to S^2−^ show a slight positive shift, demonstrating the regulated chemical environment of S atoms by Cu–Co synergistic effect [33]. In the Cu 2p spectrum (Figure 2f), typical peaks of Cu^+^ and Cu^2+^ are detected, further confirming the successful synthesis of spinel CuCo_2_S_4_. The coexistence of multivalent metal sites (Cu^+^/Cu^2+^, Co^2+^/Co^3+^) can provide abundant active centers, optimize the adsorption of reaction intermediates, and promote charge transfer, thus significantly enhancing the electrocatalytic performance in SIFBs [34,35].

To further visualize the structural evolution and atomic coordination environment during the Cu-induced topological phase transition, the schematic coordination structures of CoS_1.097_ and CuCo_2_S_4_ are illustrated in Figure 2g. In the original CoS_1.097_, S atoms are coordinated with six Co atoms in a hexagonal lattice [36]. After Cu^2+^ ion exchange and topological transformation, partial Co atoms are replaced by Cu atoms, and the coordination environment of S atoms is rearranged into an eight-coordination structure, forming a typical cubic spinel CuCo_2_S_4_ lattice [37]. In this optimized structure, Co–S bonds maintain structural stability, while the Cu–Co–S interaction provides abundant multivalent metal sites and strong electronic coupling effects, which lay a solid foundation for enhanced adsorption, accelerated charge transfer, and excellent bifunctional electrocatalytic performance in SIFBs [38,39].

Table S1 summarizes the XPS quantitative elemental results. The atomic percentages of Cu, Co, and S are 8.63 at%, 17.83 at%, and 25.62 at%, respectively, giving an atomic ratio of Cu:Co:S ≈ 1:2.07:2.97. Compared with the theoretical stoichiometry of spinel CuCo_2_S_4_ (Cu:Co:S = 1:2:4), the Co content is slightly higher than the theoretical value, while the S content is obviously deficient. Meanwhile, a high carbon content (47.91 at%) is detected, which originates from the graphite felt substrate. The deficiency of sulfur may be related to the partial oxidation of sulfur in the sample, corresponding to the characteristic peak at approximately 169 eV in the S 2p XPS spectrum. The Cu/Co atomic ratio is close to 1:2, indicating that Cu^2+^ ions have successfully replaced the Co sites in CoS_1.097_.

It should be emphasized that XPS characterizes only the surface elemental composition, and surface sulfur of metal sulfides is prone to oxidative loss, resulting in the apparent sulfur deficiency shown in Table S1. Multi-region quantitative EDX measurements were then implemented to detect bulk composition independent of graphite felt substrate. The bulk atomic ratio Cu:Co:S derived from EDX averages reaches approximately 1:2.69:4.67, with a Cu/Co stoichiometry consistent with spinel CuCo_2_S_4_ and sufficient sulfur content in the bulk (Table S2). This result is consistent with the formation of the spinel structure as revealed by XRD and the ball-and-stick model.

### 3.2. Electrocatalytic Activity and Kinetics Evaluation

**Figure 3:**
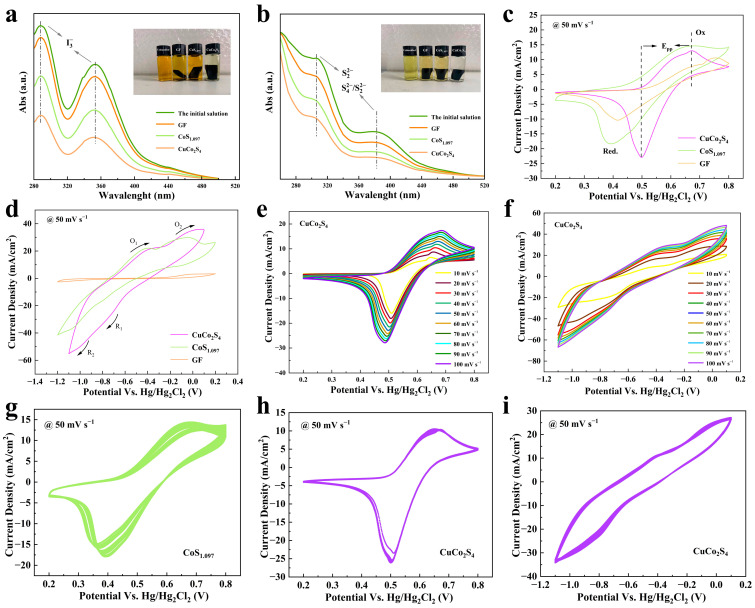
(**a**) UV–vis absorption spectra of the original solutions of NaI + I_2_ and (**b**) Na_2_S_2_, as well as the solutions after adsorption with GF, CoS_1.097_, and CuCo_2_S_4_ electrodes. Insets: optical photographs of the original solutions and the solutions after adsorption with GF, CoS_1.097_, and CuCo_2_S_4_ electrodes. CV curves of the catalysts in (**c**) 0.1 M NaI_3_ + 0.5 M NaCl solution and (**d**) 0.06 M Na_2_S + 0.02 M S + 0.5 M NaCl solution at a scan rate of 50 mV s^−1^. CV curves of CuCo_2_S_4_ (**e**) in 0.06 M Na_2_S + 0.02 M S + 0.5 M NaCl solution and (**f**) in 0.1 M NaI_3_ + 0.5 M NaCl solution at various scan rates. CV curves of (**g**) CuCo_2_S_4_ in 0.06 M Na_2_S + 0.02 M S + 0.5 M NaCl solution, (**h**) CuCo_2_S_4_ and (**i**) CoS_1.097_ in 0.1 M NaI_3_ + 0.5 M NaCl solution at various scan rates.

The adsorption capability of the electrode toward electroactive species is a key factor affecting the electrocatalytic performance. Visual adsorption tests and UV–vis spectroscopy were employed to evaluate the adsorption affinity of GF, CoS_1.097_, and CuCo_2_S_4_ for I_3_^−^ and S_2_^2−^ species, as presented in Figure 3a,b. For the iodine-containing solution (NaI + I_2_), the absorbance peak intensity decreases slightly after interaction with GF and CoS_1.097_, and the solution color shows no obvious change. In sharp contrast, the absorption peak declines dramatically, and the solution becomes nearly colorless after adsorption by CuCo_2_S_4_, demonstrating significantly enhanced adsorption capability for polyiodide species [16]. Similarly, in the polysulfide solution (Na_2_S_2_), CuCo_2_S_4_ also exhibits much stronger adsorption toward S_2_^2−^ than GF and CoS_1.097_. The excellent adsorption performance benefits from the abundant active sites induced by the bimetallic synergy and the open nanoflower structure, which can effectively capture polysulfide and polyiodide intermediates, thus accelerating redox kinetics [9,40].

To further evaluate the ability of as-prepared electrodes to suppress the shuttle effect, H-cell static crossover tests were implemented. The separator used in the H-cell was Nafion N117 membrane, which is exactly the same as the membrane assembled in the practical flow battery. Figure S1 displays the digital photographs of different groups after 12 h of static placement. For the Na_2_S_2_ system (Figure S1a), the solution loaded with CuCo_2_S_4_ turned nearly colorless. In comparison, the solution with CoS_1.097_ only presented slight color fading, and the blank graphite felt (GF) group had no visible change. A similar phenomenon was observed in the NaI + I_2_ system (Figure S1b). These phenomena indicate that CuCo_2_S_4_ can strongly adsorb polysulfide and I_3_^−^/I^−^ intermediates on the electrode surface. It greatly reduces the content of free active ions in the electrolyte, and further inhibits the crossover of redox species through the membrane from cathode to anode and vice versa. This is a crucial reason for the high Coulombic efficiency and long cycling stability of the assembled flow battery.

To quantitatively verify that the nanoflower-like architecture increases the active surface area and accessible active sites, we calculated the electrochemical active surface area (ECSA) via double-layer capacitance (C_dl_) obtained from cyclic voltammetry curves at various scan rates. Among them, the double-layer capacitance Cdl is determined according to the slope of the curve. The ordinate Δi represents the difference between the anodic current density and the cathodic current density, and the abscissa ν is the potential scanning rate (mV·s^−1^). We believe that the value of ECSA is the ratio of the double-layer capacitance (Cdl) to the specific capacitance constant Cs. The specific capacitance constant can be obtained according to the relevant formula:

$$C_{s}=\frac{Q}{2\cdot ∆V\cdot m}$$

where *Q* represents the passed charge, Δ*V* is the measured potential range, and *m* is the mass of the active material [41].

Generally, a larger C_dl_ value corresponds to a higher ECSA and more exposed active sites. Figures S2 and S3 display the linear fitting curves of current density versus scan rate for CuCo_2_S_4_ and CoS_1.097_ in polysulfide and iodide electrolytes, respectively. The calculated ECSA values are summarized in Figures S2d and S3d. In the polysulfide system, the ECSA of CuCo_2_S_4_ is 11.86 mF cm^−2^, which is obviously higher than 8.72 mF cm^−2^ of CoS_1.097_. In the iodide system, CuCo_2_S_4_ delivers an ECSA of 23.84 mF cm^−2^, while the value of CoS_1.097_ is only 5.52 mF cm^−2^. The above results clearly indicate that the open nanoflower structure of CuCo_2_S_4_ can greatly enlarge the electrochemical active surface area and provide numerous accessible active sites, which is favorable for accelerating the redox kinetics of polysulfide and iodide couples.

Electrocatalytic activity and redox reversibility were further investigated by cyclic voltammetry (CV). Figure 3c,d shows the CV curves of different electrodes in iodine and polysulfide electrolytes. In the I^−^/I_3_^−^ electrolyte, CuCo_2_S_4_ displays a pair of well-defined redox peaks with the highest current density and the smallest peak-to-peak potential separation (E_pp_) of only 0.174 V, much lower than 0.287 V for CoS_1.097_ and 0.367 V for GF (Table S3). Meanwhile, the ratio of |J_(Red)_/J_(ox)_| reaches 1.77, indicating superior redox reversibility [10]. In the S^2−^/S_x_^2−^ electrolyte, CuCo_2_S_4_ also exhibits stronger response current and more reversible redox peaks compared with CoS_1.097_ and GF. These results confirm that CuCo_2_S_4_ serves as an efficient bifunctional electrocatalyst that can remarkably boost the reaction kinetics of both I^−^/I_3_^−^ and S^2−^/S_x_^2−^ redox couples [9,14].

To further verify the Cu–Co bimetallic synergy and exclude the dominant contribution of nanoflower morphology, we synthesized pure copper sulfide (Cu_9_S_5_) under the identical preparation conditions as a supplementary control material. Figure S4 presents the XRD pattern of the as-prepared sample. All diffraction peaks match well with the standard card of Cu_9_S_5_ (PDF#47-1748), confirming the successful synthesis of pure copper sulfide. Figure S5a shows the CV curves of Cu_9_S_5_ measured at the scan rate of 50 mV·s^−1^ under the same test conditions. In the polysulfide system, only a single reduction peak is observed for Cu_9_S_5_, and its current density is lower than that of CuCo_2_S_4_. This indicates that Cu_9_S_5_ has a weak capacity to drive the conversion of polysulfides and cannot facilitate the complex multi-electron reactions, resulting in insufficient catalytic activity. In the iodide system, Cu_9_S_5_ delivers a larger E_pp_ value than CuCo_2_S_4_ (Figure S5b). A larger E_pp_ stands for worse redox reversibility and slower charge transfer kinetics. Overall, Cu_9_S_5_ exhibits much poorer performance than CuCo_2_S_4_. These results confirm that the Cu–Co bimetallic synergy formed by introducing Co elements can effectively improve the catalytic activity of the material.

To further explore the kinetic features, CV tests at various scan rates were conducted, as shown in Figure 3e,f. The redox peak currents increase linearly with the square root of the scan rate, revealing that the reactions are diffusion-controlled processes [14,21]. Meanwhile, the CV curves retain good similarity without obvious distortion even at high scan rates, verifying the structural stability and rapid charge transport property of CuCo_2_S_4_. Furthermore, long-term cycling CV tests were performed to evaluate stability (Figure 3g–i). After 200 consecutive cycles, the CV curve of CuCo_2_S_4_ almost overlaps with the initial curve, whereas CoS_1.097_ shows obvious signal attenuation. The nearly coincident CV curves before and after long-term cycling reveal that the catalyst possesses stable electrocatalytic performance and reliable repeatability. The excellent cycling stability originates from the robust spinel structure and the stable interfacial interaction between the catalyst and graphite felt, which is critical for long-term operation in SIFBs.

### 3.3. Electrochemical Performance of SIFB Batteries

**Figure 4:**
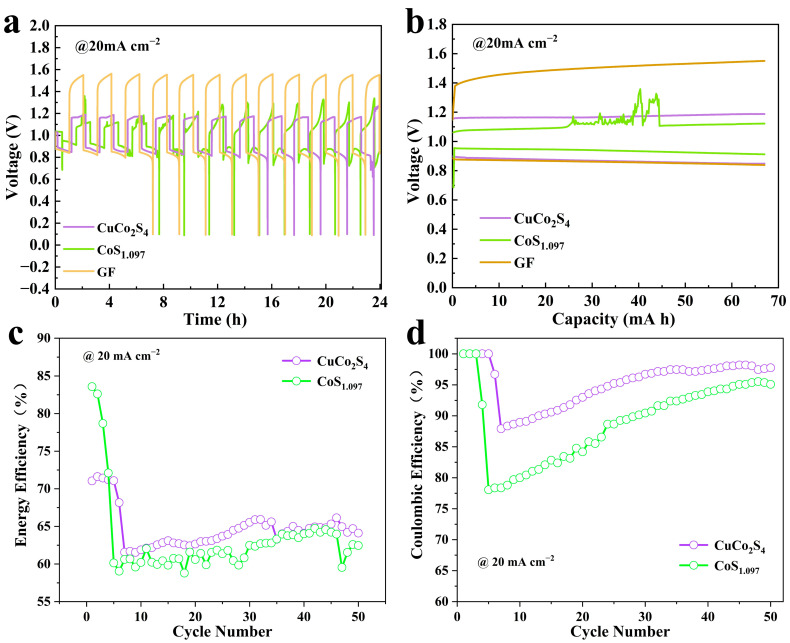
(**a**) Polarization curves and (**b**) initial galvanostatic charge–discharge voltage-capacity profiles of various electrodes at a current density of 20 mA·cm^−2^. (**c**) Energy efficiency and (**d**) Coulombic efficiency of various electrodes at 20 mA·cm^−2^ for 50 cycles.

The electrochemical performance of as-prepared electrodes in SIFBs was systematically evaluated using static polarization, galvanostatic charge–discharge, cycling stability, and electrochemical impedance spectroscopy (EIS). Figure 4a presents the polarization curves at 20 mA·cm^−2^. The blank GF electrode shows the largest voltage gap, indicating severe polarization and poor intrinsic activity. The CoS_1.097_ electrode exhibits improved performance but undergoes rapid degradation during cycling. In sharp contrast, the CuCo_2_S_4_ electrode displays a much smaller voltage gap and a stable polarization profile, demonstrating significantly reduced electrochemical polarization and accelerated reaction kinetics [31].

Figure 4b shows the initial galvanostatic charge–discharge curves at 20 mA cm^−2^. The GF electrode presents a voltage gap as high as 0.64 V, while the CuCo_2_S_4_ electrode delivers a remarkably small voltage gap of only 0.29 V with flat and stable charge–discharge plateaus. The CoS_1.097_ electrode shows obvious voltage fluctuations, implying poor structural stability and unfavorable reaction reversibility [15]. The superior voltage performance of CuCo_2_S_4_ can be attributed to the bimetallic synergistic effect, favorable adsorption behavior, and rapid interfacial charge transfer [42], which collectively enhance the redox kinetics of both I^−^/I_3_^−^ and S^2−^/S_x_^2−^ couples.

Long-term cycling performance was then evaluated at 20 mA cm^−2^ for 50 cycles, as shown in Figure 4c,d. After the activation process, the CuCo_2_S_4_ electrode exhibits a highly stable energy efficiency (EE) of 62–66% with negligible decay, and the coulombic efficiency (CE) increases continuously to nearly 98%. In contrast, the CoS_1.097_ electrode shows rapid EE decay and low CE values throughout cycling. The outstanding cycling stability confirms that the spinel CuCo_2_S_4_ can effectively inhibit structural degradation and side reactions, thus maintaining robust catalytic activity during long-term operation.

**Figure 5:**
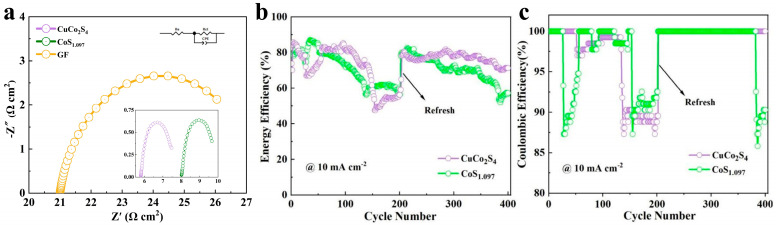
(**a**) Electrochemical impedance spectra of different electrodes. (**b**) Energy efficiency and (**c**) Coulombic efficiency of various electrodes for long-term cycling at 10 mA cm^−2^.

EIS measurements were carried out to reveal the electrode kinetics and interfacial resistance. As displayed in Figure 5a, all Nyquist plots exhibit a typical semicircle in the high–medium frequency region related to charge transfer resistance (R_ct_). The CuCo_2_S_4_ electrode shows the smallest semicircle diameter, corresponding to an extremely low R_ct_ value of 1.75 Ω cm^2^, which is much lower than those of GF and CoS_1.097_. The significantly reduced R_ct_ demonstrates faster charge transfer and superior electrocatalytic kinetics [43], which is highly consistent with the enhanced battery performance.

To further examine the ultra-long stability, 400-cycle tests were performed at 10 mA·cm^−2^ with an electrolyte refresh at the 200th cycle (Figure 5b,c). After refreshing the electrolyte, both electrodes exhibit recovered performance, confirming that the performance degradation is mainly caused by electrolyte consumption rather than irreversible structural collapse of the catalyst [44]. Notably, the CuCo_2_S_4_ electrode maintains a high energy efficiency above 70% after 400 cycles, while CoS_1.097_ undergoes rapid efficiency fading. The remarkable stability and recoverability demonstrate the great potential of CuCo_2_S_4_ for practical long-duration SIFB applications.

To intuitively confirm the structural robustness of the nanoflower-like CuCo_2_S_4_ after prolonged cycling, SEM characterization was carried out. As shown in Figure S7, the electrode still maintains a complete open nanoflower structure after long-term operation on the anodic polysulfide side, without obvious damage or collapse of the nanosheet assembly. Such excellent morphological preservation accounts for the stable electrocatalytic activity and high energy efficiency of the CuCo_2_S_4_ electrode during hundreds of charge–discharge cycles.

To comprehensively evaluate the performance of the nanoflower-like CuCo_2_S_4_ bifunctional electrocatalyst, we summarize the series resistance, energy efficiency and cycle stability of recently reported catalysts for polysulfide/iodide redox flow batteries in Table 1 [11,16,45,46]. At 20 mA·cm^−2^, CuCo_2_S_4_ achieves a high energy efficiency of 66% and a low series resistance of 5.80 Ω cm^2^, accompanied by an ultra-long cycle lifespan of 400 cycles. Although a few reported materials deliver slightly higher energy efficiency or smaller impedance individually, most of them suffer from larger ohmic resistance, lower working current density or much shorter cycle durability [47,48]. It can be concluded that the as-prepared CuCo_2_S_4_ realizes a favorable balance of low impedance, high energy efficiency and outstanding long-term cycling stability compared with other reported counterparts (see Table 1).

## 4. Conclusions

In conclusion, an ultrathin nanoflower-like spinel CuCo_2_S_4_ bifunctional electrocatalyst was successfully synthesized on GF via a facile hydrothermal-sulfurization method, and its application in SIFBs was systematically studied. Structural characterizations confirmed the successful phase transition from hexagonal CoS_1.097_ to cubic spinel CuCo_2_S_4_, with a homogeneous elemental distribution and Cu/Co atomic ratio close to 1:2. The unique nanoflower architecture provides abundant active sites and open channels for mass transport, while the bimetallic synergistic effect (Cu^+^/Cu^2+^ and Co^2+^/Co^3+^) enhances adsorption toward polyiodide/polysulfide species and accelerates redox kinetics. Electrochemical tests demonstrated that CuCo_2_S_4_ exhibits a small peak-to-peak potential separation (0.174 V for I^−^/I_3_^−^) and low charge transfer resistance (1.75 Ω·cm^2^). The assembled SIFB delivers a narrow voltage gap of 0.29 V at 20 mA·cm^−2^, stable energy efficiency of 62–66% over 50 cycles, and excellent long-term stability (≥70% efficiency after 400 cycles). This work provides a feasible strategy for designing high-performance bifunctional electrocatalysts via bimetallic spinel structure and morphology optimization, promoting the development of low-cost, long-duration SIFBs for grid-scale energy storage.

## Figures and Tables

**Table 1:** Performance comparison between the as-prepared CuCo_2_S_4_ and previously reported bifunctional electrocatalysts for polysulfide/iodide redox flow batteries.

Catalyst	R_s_ (Ω cm^2^)	Energy Efficiency (%)	Cycle Life	Reference
CuCo_2_S_4_	5.80	66%@20 mA·cm^−2^	400 cycles	This Work
KB/S-HCN-2:1-CF	2.58	40.9%@40 mA·cm^−2^	200 cycles	[11]
Cu_7_S_4_-SKE	11.06	77.2%@20 mA·cm^−2^	200 cycles	[16]
Cu_2_CoGeS_4_	11.32	50%@40 mA·cm^−2^	80 cycles	[44]
Cu_7_S_4_/CNT	8.10	66%@30 mA·cm^−2^	500 h	[45]

## Data Availability

The original contributions presented in this study are included in the article/Supplementary Materials. Further inquiries can be directed to the corresponding authors.

## Supplementary Materials

The following supporting information can be downloaded at: https://www.mdpi.com/article/10.3390/ma19132839/s1, Figure S1: Digital photographs of H-cell crossover tests after 12 h static state. (a) Na_2_S_2_ solution system. (b) NaI + I_2_ solution system; Figure S2: (a,b) CV curves of CuCo_2_S_4_ and CoS_1.097_ at different scan rates in polysulfide electrolyte (0.06 M Na_2_S + 0.02 M S + 0.5 M NaCl). (c) Linear fitting of capacitive current against scan rate. (d) ECSA histogram of the two samples; Figure S3: (a,b) CV curves of CuCo_2_S_4_ and CoS_1.097_ at different scan rates in iodide electrolyte (0.1 M NaI_3_ + 0.5 M NaCl). (c) Linear fitting of capacitive current against scan rate. (d) ECSA histogram of the two samples; Figure S4: XRD pattern of Cu_9_S_5_; Figure S5: Electrochemical cyclic voltammetry curves of Cu_9_S_5_ with a scan rate of 50 mV s^−1^ in (a) 0.06 M Na_2_S + 0.02 M S + 0.5 M NaCl solution and (b) 0.1 M NaI_3_ + 0.5 M NaCl solution; Figure S6: Optical photograph of the assembled single polysulfide/iodide redox flow cell; Figure S7: SEM images of the CuCo_2_S_4_ electrode after long-term cycling on the polysulfide anolyte side; Table S1: Atomic percentages of Cu, Co, S, and C elements in CuCo_2_S_4_ obtained by XPS analysis; Table S2: Atomic percentages of Cu, Co and S elements in CuCo_2_S_4_ obtained by EDX analysis; Table S3: Parameters of CV curves of different electrodes in I^−^/I_3_^−^ solution.
