# Supplementary material for: Nanoflower-like CuCo2S4 with Bimetallic Synergy as High-Performance Bifunctional Electrocatalyst for Polysulfide/Iodide Redox Flow Batteries
Source: Materials (Basel). 2026 Jul 3;19(13):2839. doi: 10.3390/ma19132839 (PMC13362695; doi:10.3390/ma19132839)
Supplement: Supplementary file 1 [file materials-19-02839-s001.zip › materials-4352418-supplementary.pdf]

Supporting Information for

Nanoflower-like $CuCo_2S_4$ with Bimetallic Synergy as High-Performance Bifunctional Electrocatalyst for Polysulfide/Iodide Redox Flow Batteries

Shuo Liu[a1], Renyi Wei[b1], Jingwen Zhang[b], Xiaoxin Dan[b], Mingying Chen[b], Wenxian Liu[c]*, Jia He[d], and Xijun Liu[b]**

[a] *School of Energy and Chemical Engineering, Tianjin Renai College, Tianjin 301636, China*

[b] *MOE Key Laboratory of New Processing Technology for Nonferrous Metals and Materials, Guangxi Key Laboratory of Processing for Non-ferrous Metals and Featured Materials, School of Resources, Environment and Materials, Guangxi University, Nanning, Guangxi 530004, China*

[c] *College of Materials Science and Engineering, Zhejiang University of Technology, Hangzhou, 310014 Zhejiang, China*

[d] *Institute for School of Chemistry and Chemical Engineering, Tianjin University of Technology, Tianjin 300384, China*

***Corresponding authors:** *jwaaaaaaa_22@163.com;* liuwx@zjut.edu.cn; xjliu@gxu.edu.cn.

[1] These authors have contributed equally to this work.

Experimental

Materials and Reagents

All chemical reagents were of analytical grade and used as received without further purification. Cobalt nitrate hexahydrate ($Co(NO_3)_2 \cdot 6H_2O$), copper nitrate ($Cu(NO_3)_2$), thiourea (CH_4N_2S), sublimed sulfur (S), sodium iodide (NaI), iodine (I_2), sodium disulfide (Na_2S_2), sodium chloride (NaCl), ethanol, and Nafion solution (5 wt%) were purchased from commercial suppliers (Shanghai Macklin Biochemical Co., Ltd.). Deionized water was used throughout all experiments. Graphite felt (GF, 3 mm thickness) was used as the electrode substrate (Beijing Jinglong Special Carbon Technology Co., Ltd.).

Characterization.

X-ray diffraction (XRD) patterns were collected on a Rigaku SMARTLAB 3 kW diffractometer with Cu Kα radiation. Scanning electron microscopy (SEM) images were obtained using a Hitachi SU8020 microscope. Transmission electron microscopy (TEM) and high-resolution TEM (HRTEM) images were captured on a FEI Titan G2 ETEM microscope. Energy-dispersive X-ray (EDX) elemental mapping was performed to analyze the element distribution. X-ray photoelectron spectroscopy (XPS) measurements were conducted on a Thermo Fisher K-Alpha 1063 spectrometer. All binding energies were calibrated using the C 1s peak at 284.8 eV. UV-vis absorption spectra were recorded on a UV-1800PC spectrophotometer for visualization adsorption tests.

Detailed information of flow cell and N117 membrane.

The separator used in this work is Nafion N117 membrane (Suzhou Shengnuo Technology Co., Ltd.). The membrane thickness is 183 μm (7.2 mil), with an ion exchange capacity (IEC) of 0.89–0.90 meq/g and an area specific resistance (ASR) of 0.18–0.22 $\Omega \cdot cm^2$.

The pretreatment process for N117 membrane is as follows: (1) Soak the membrane in 5 wt% H_2O_2 solution at 80 °C for 1 h; (2) Transfer the membrane to 5 wt% H_2SO_4 solution and keep it at 80 °C for 1 h; (3) Immerse the membrane in 1 M NaOH solution at 80 °C for 2 h. The membrane was rinsed with deionized water for 10 min after each above treatment step. The active electrode area was 2 cm × 2 cm. The single cell device was assembled with ion exchange membrane, bipolar plates, current collectors, positive/negative electrodes and electrode frames. The optical photograph of the assembled flow cell is shown in Figure S6.

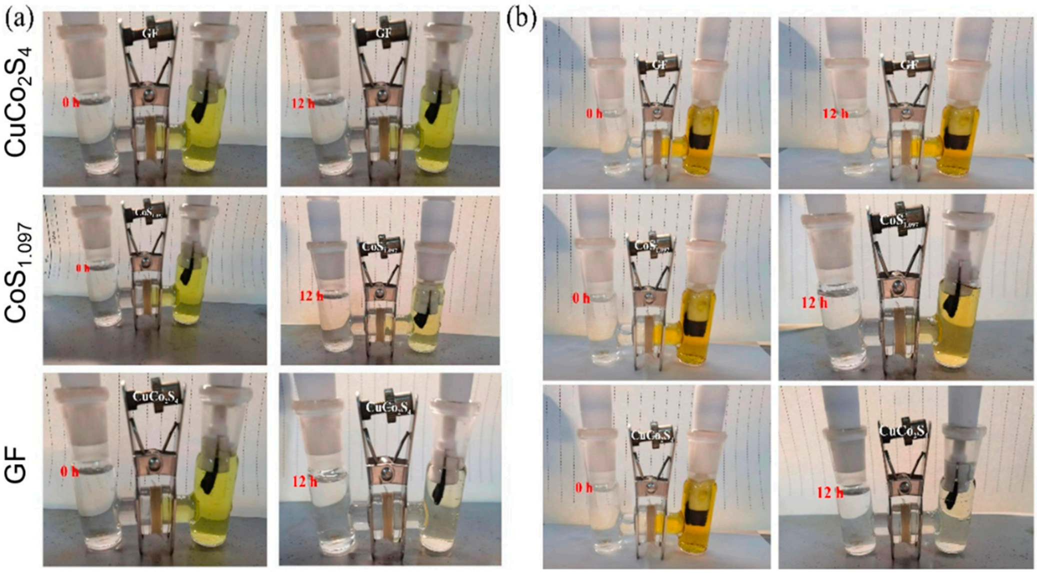

Figure S1 Digital photographs of H-cell crossover tests after 12 h static state. (a) Na_2S_2 solution system. (b) $NaI+I_2$ solution system.

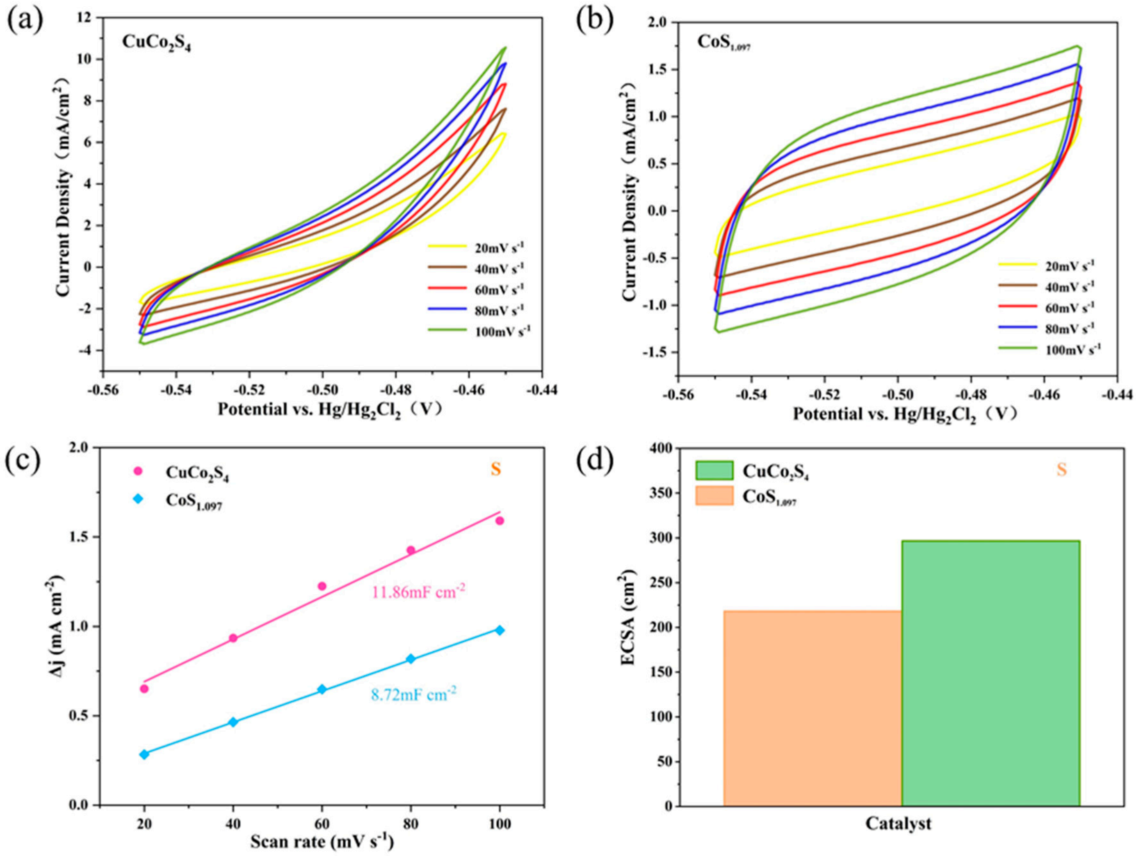

Figure S2 (a, b) CV curves of $CuCo_2S_4$ and $CoS_{1.097}$ at different scan rates in polysulfide electrolyte (0.06 M Na_2S + 0.02 M S + 0.5 M NaCl). (c) Linear fitting of capacitive current against scan rate. (d) ECSA histogram of the two samples.

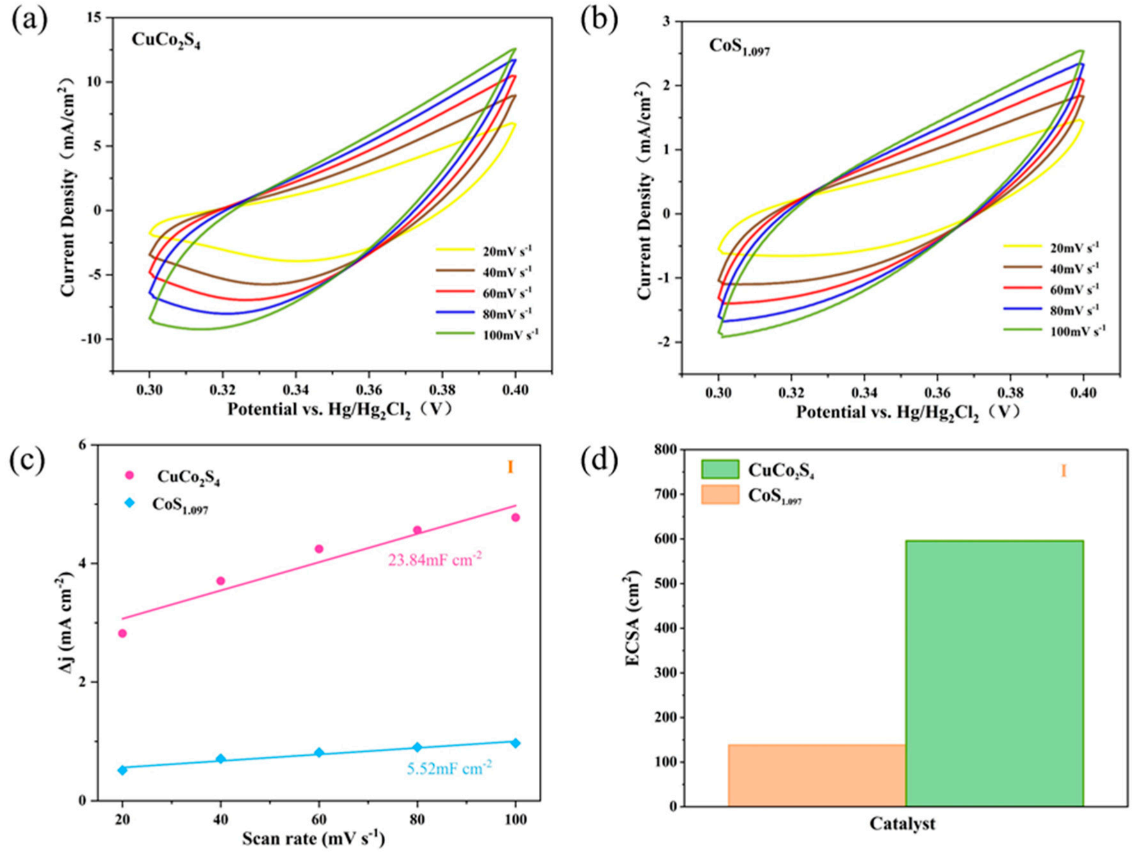

Figure S3 (a, b) CV curves of $CuCo_2S_4$ and $CoS_{1.097}$ at different scan rates in iodide electrolyte (0.1 M NaI_3 + 0.5 M NaCl). (c) Linear fitting of capacitive current against scan rate. (d) ECSA histogram of the two samples.

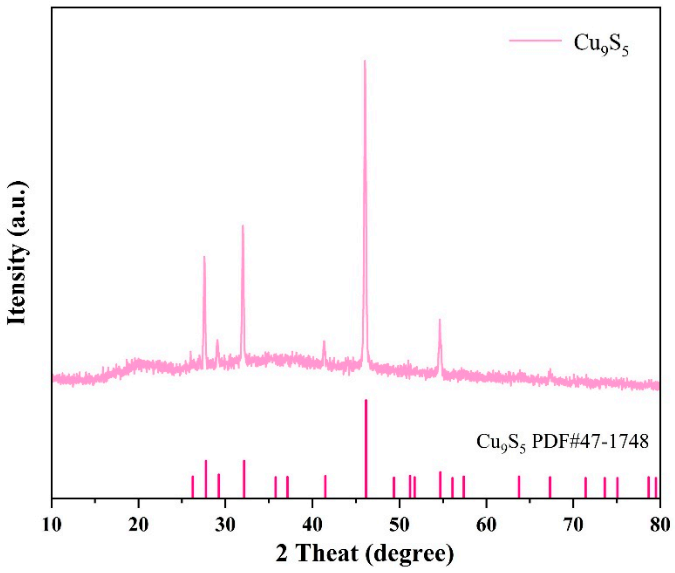

Figure S4 XRD pattern of Cu_9S_5.

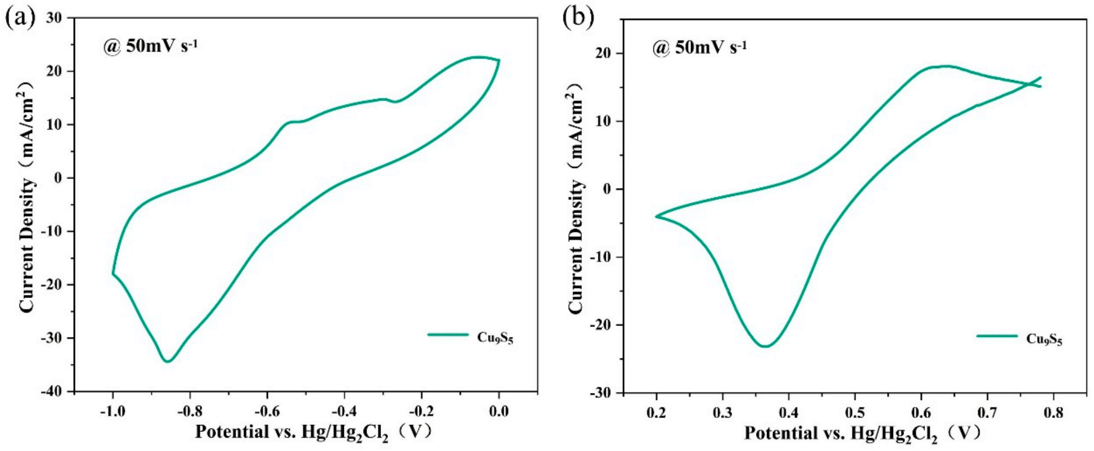

Figure S5 Electrochemical cyclic voltammetry curves of Cu_9S_5 with a scan rate of 50 mV s^{-1} in (a) 0.06 M Na_2S + 0.02 M S + 0.5 M NaCl solution and (b) 0.1 M NaI_3 + 0.5 M NaCl solution.

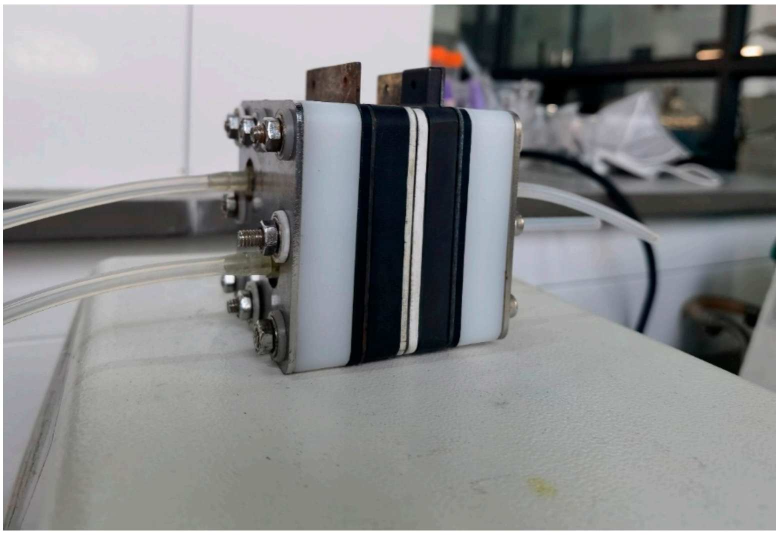

Figure S6 Optical photograph of the assembled single polysulfide/iodide redox flow cell.

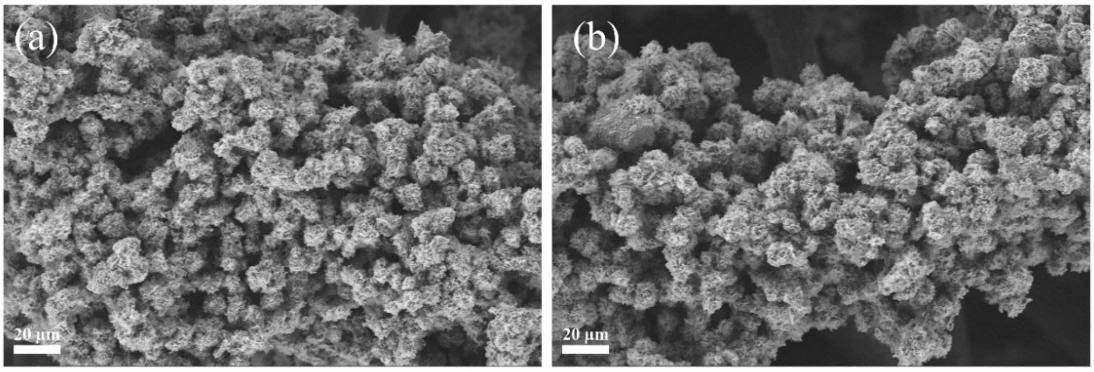

Figure S7 SEM images of the $CuCo_2S_4$ electrode after long-term cycling on the polysulfide anolyte side.

Table S1 Atomic percentages of Cu, Co, S, and C elements in $CuCo_2S_4$ obtained by XPS analysis.

Sample	Cu (at%)	Co (at%)	S (at%)	C (at%)
$CuCo_2S_4$	8.63	17.83	25.62	47.91

Table S2 Atomic percentages of Cu, Co and S elements in $CuCo_2S_4$ obtained by EDX analysis.

Type of element	Atomic percentage of Region 1 (%)	Atomic percentage of Region 2 (%)	Average value (%)
Cu	10.52	13.40	11.96
Co	30.52	33.94	32.23
S	56.96	54.66	55.81

Table S3 Parameters of CV curves of different electrodes in I^-/I_3^- solution.

Samples	J_{ox} [mA cm^{-2}]	J_{Red} [mA cm^{-2}]	$\lvert J_{Red}/J_{ox} \rvert$	E_{ox} [v]	E_{red} [v]	E_{pp} [v]
GF	10.18	-9.80	0.96	0.754	0.387	0.367
$CoS_{1.097}$	14.64	-18.36	1.25	0.679	0.392	0.287
$CuCo_2S_4$	12.98	-22.92	1.77	0.673	0.499	0.174
